# Bioaccessibility and Intestinal Transport of Deltamethrin in Pacific Oyster (*Magallana Gigas*) Using Simulated Digestion/NCM460 Cell Models

**DOI:** 10.3389/fnut.2021.726620

**Published:** 2021-08-17

**Authors:** Yadan Jiao, Chune Liu, Chunsong Feng, Joe M. Regenstein, Yongkang Luo, Yuqing Tan, Hui Hong

**Affiliations:** ^1^Beijing Laboratory for Food Quality and Safety, College of Food Science and Nutritional Engineering, China Agricultural University, Beijing, China; ^2^Institute of Yantai, China Agricultural University, Yantai, Shandong, China; ^3^Department of Food Science, Cornell University, Ithaca, NY, United States

**Keywords:** deltamethrin, bioaccessibility, oyster, NCM460 cells, *magallana gigas*

## Abstract

Deltamethrin (DEL) can be introduced into the food chain through bioaccumulation in Pacific oysters, and then potentially threaten human health. The objective of this study was to investigate the bioaccessibility of DEL in oysters with different cooking methods after simulated digestion. DEL content in different tissues of oysters going from high to low were gills, mantle, viscera, and adductor muscle. Bioaccessibility of DEL in oysters decreased after steaming (65%) or roasting (51%) treatments compared with raw oysters (82%), which indicated that roasting can be used as a recommended cooking method for oysters. In the simulated digestion process, the concentration of DEL in the digestive juice and the bioaccessibility of DEL were affected by the pH in the gastric phase. And the transport efficiency of DEL through the monolayer molecular membrane of NCM460 cells ranged from 35 to 45%. These results can help assess the potential harm to consumers of DEL in shellfish. Furthermore, it provides a reference for the impact of lipophilic toxins in seafood.

## Highlights

- The content of deltamethrin is related to the interaction of protein and fat.
- The bioaccessibility of oysters decreased after steaming and roasting.
- Cooking treatment may result in the conversion of deltamethrin isomers.
- The pH of gastric phase will affect the bioaccessibility of deltamethrin in oysters.

## Introduction

Synthetic pyrethroids are broad-spectrum, efficient, and neurotoxic pesticides. Pyrethroid use markedly increased and became the preferred choice in many agriculture-based countries in the last two decades since the implementation of restrictions on the sale of organophosphorus insecticides (1, 2). Pyrethroids have high insecticidal potency but they have a low toxicity with birds and mammals (3). With mollusks, previous studies have reported that pyrethroids were more toxic to aquatic animals than to mammals because of their lower ability to degrade pyrethroid pesticides (4, 5). Therefore, pyrethroids are generally not directly used in aquaculture. However, there are some reports of pyrethroid pesticide residues in aquatic products and their habitat (6–8).

Given its widespread and worldwide use, pyrethroid pollution has become an increasing threat to human health (9). There are many pathways for the accumulation of pyrethroids in mollusks. One is through water pollution caused by the use of pyrethroids to kill water-borne parasites. Another is the run-off from the use of pyrethroids in agricultural or urban areas (10, 11). Pyrethroids in the aquaculture environment not only has a negative impact on aquatic organisms but also threatens consumer's health (12–15).

Deltamethrin (DEL), a type II synthetic pyrethroid, is used widely as an insecticide (16, 17). It was used in agricultural and aquaculture for pest control (18). Some studies have detected the metabolites of DEL in the urine of adults, pregnant women, and children (19, 20). Skin contact and oral intake of DEL-contaminated food are harmful to human health (21). Several studies found various toxic effects in humans, such as neurotoxicity, immunotoxicity, and reproductive toxicity (22–24).

Oysters are becoming more widely consumed because of their appreciable quantities of proteins, long-chain polyunsaturated fatty acids, vitamins and minerals, and desirable sweet and “umami” tastes (25). Oysters are a filter-feeding shellfish. Because they filter large quantities of water, microorganisms and other pollutants will accumulate in tissue (25, 26). These pollutants will enter the human body through consumption. The maximum residue limit of DEL is 0.5 mg/kg in mammals, but there are no standards for aquatic products (27). Oysters are often consumed raw. However, raw oysters may contain many health-threatening factors including *Vibrio cholera* and enteropathogenic bacteria (28–30). Cooking can eliminate or reduce some harmful substances in oysters (31). But the effect of cooking on DEL remains unknown. Moreover, the amount of a contaminant in a food as purchased is not necessarily equal to how much human body will absorb. After DEL contaminated food enters the human body, it is digested in the gastrointestinal tract. Part of the DEL will enter the body with the absorption of the digestive juices, while the rest if undigested will be excreted with the digestive residue. There is, however, no previous study reported on the bioaccessibility of DEL in any food matrix in the human body. In recent years, *in vitro* models have been widely used in the study of bioaccessibility (32, 33). Understanding the bioaccessibility of DEL in the human body can provide a preliminary estimate of its bioavailability data, which can assess the threat of DEL to human health more accurately. The bioavailability data can also provide a reference for a risk assessment of DEL in seafood.

*In vitro* simulated digestion and NCM460 cell model are two common *in vitro* models. Simulated digestion is used to simulate the physiological environment of human gastrointestinal tract to study the physical and chemical changes that may occur in the process of gastrointestinal digestion after food intake, the interaction between food and digestive fluid and food in the process of digestion, the utilization rate of nutrients and the metabolism of toxic substances (34). The normal human colon-derived mucosal epithelial cell line (NCM460) is a common cell model. It can be used to study the absorption and transportation of nutrients or harmful substances in drugs and food through the intestine. In this study, the bioaccessibility of deltamethrin in oyster was studied by simulated digestion and NCM460 cell experiment.

Thus, the purpose of this study was to: (1) investigate the enrichment of deltamethrin in different tissues of oyster; (2) assess the bioaccessibility of the DEL in oysters cooked with different methods (teaming and roasting) after simulated digestion; (3) study the penetration of DEL through intestinal epithelial cells.

## Materials and Methods

### Collection of Oysters and Sample Preparation

Oyster samples were collected from the border of the Bohai and the Yellow seas (Weihai, Shandong Province, China), and all the oysters were transported to the laboratory alive. After that, the oysters were acclimated in artificial seawater (32 g/L) for 24 hrs. The seawater was produced using seawater crystals (Tianjin Binhai New Area Tanggu Hai Sheng Seawater Crystal Factory, Tianjin, China) according to Bielmyer et al. (35). Then, the oysters were exposed to seawater with DEL (2 μg /L) for 72 hrs. DEL for contamination was obtained from Bayer CropScience (Hangzhou, Zhejiang Province, China). Salinity (27.0 ± 1.0‰) and water temperature (18 ± 1°C) were maintained throughout. Photoperiod at 12 h light/12 h dark was maintained. To prevent the residual chlorine in the tap water from affecting the oyster culture process, the seawater was oxygenated for 48 hrs before use.

All the artificially contaminated oysters were divided into four groups, of which three groups were subjected to different processing methods: raw, steamed (i.e., steaming in an steam cooker for 5 min, until the oyster shell opens), and roasted (i.e., in an oven at 200°C for 20 min, only use downfire). Oyster tissue was taken out and divided into four parts: gill, viscera, mantle, and adductor muscle. Twenty-five specimens were removed from the treated oysters. For all samples, the tissue was separated from the oyster-shell, cleaned with running water. The collected tissues were then drained and homogenized with a blender (JY-200B, Zhongshan Jiuyuan Electric Appliance Co. Ltd, Zhongshan, Guangdong Province, China) until visually homogenized. These samples were stored at −20°C for further analyses (maximum 4 weeks of storage).

### *In vitro* Digestion Model

**Figure 1:**
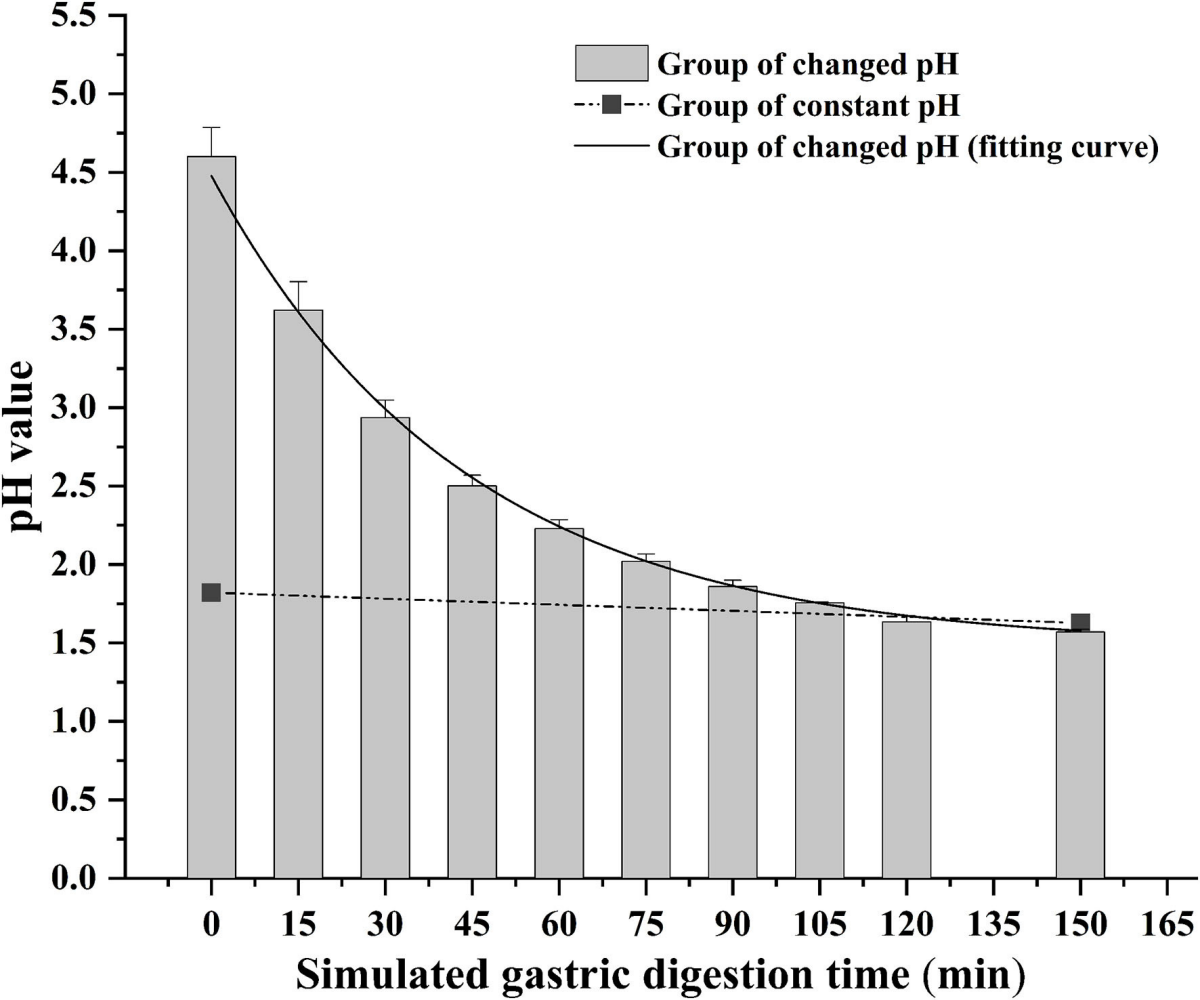
Change of gastric pH during simulated digestion. The solid line is the fitted curve obtained for the pH varied group.

The bioaccessibility of DEL in oysters was assessed using an *in vitro* digestion INFOGEST procedure previously described by Brodkorb et al. (36) with proper modifications. In short, the simulated human digestion methodology was carried out in three different phases (oral, gastric, and intestinal) using four digestive enzymes: pepsin (Aladdin, P110927), lipase (Aladdin, L299012), trypsin from swine (Sigma, P7545) and cow bile (Solarbio, B8210). And each oyster sample was digested in triplicate. For each sample, 5 g of oyster tissue was digested at 37°C using a constant temperature water bath shaker (JieRuiEr THZ-82; Changzhou, Jiangsu Province, China). The simulated digestion was done using the following protocol: oral phase (the tissue is diluted 1:1 with simulated salivary fluid at pH 7 ± 0.2; 2 min), gastric phase (the oral bolus is diluted 1:1 with simulated gastric fluid; 150 min), and intestinal phase (the gastric chyme is diluted 1:1 with simulated intestinal fluid at pH 7 ± 0.2; 120 min). Studies have shown that the pH in the gastric phase is constantly changing during the digestion process (37). To better simulate the real physiological environment during the gastric digestion process, the pH is continuously reduced by adding hydrochloric acid (the total amount of hydrochloric acid added is 2 mL, 0.4 mL is added at the beginning of the gastric phase, and then 0.2 mL is added every 15 min) during the experiment. The change of pH in the gastric phase is shown in Figure 1. Each simulated digestion fluid was prepared just before simulated digestion to avoid the loss of enzymatic activity. At the end of the simulated digestion, the pH of gastric samples was adjusted to 8.0 ± 0.2 and the intestinal samples were heated in the water bath at 95°C for 10 min to stop the digestion process. After that, the digested samples were centrifuged at 2,930 × g at 4°C for 5 min to separate the bioaccessible and non-bioaccessible fractions.

Considering that the content of DEL in the digestive phase is changing, the steamed oyster group (one of the most common cooking method of oysters) was selected to study the concentration changes of DEL in cooked oysters during digestion. Samples were taken every 30 min during the simulated digestion process for DEL analysis.

### DEL Analysis

#### Reagents

All reagents used for DEL extraction and analysis were of analytical grade or higher. Acetonitrile (p.a. ≥99.9%) and N-hexane (GC grade) were obtained from Aladdin. The QuEChERS kits (MQ3-3) were obtained from Shandong Qingyun Experiment Material Co., Ltd. (Yantai, Shandong, China); and glacial acetic acid (p.a. ≥99.9%) was obtained from Sinopharm Chemical Reagent Co., Ltd. (Shanghai, China). Ultrapure water was obtained using a Milli-Q Reference system from Millipore (Billerica, MA, USA). A certified reference standard solution of DEL was purchased from ANPEL Laboratory Technologies Inc. (Shanghai, China), which had been provided by the Agro-Environmental Protection Institute, Ministry of Agriculture and Rural Affairs of China. Calibration curves of deltamethrin-1 and deltamethrin-2 were prepared at different concentrations ranging between 1 and 40, and 10 and 400 ng mL^−1^, respectively.

#### DEL Extraction

The DEL was extracted using QuEChERS kits. The kits consisted of an extraction tube (a 50 mL plastic centrifuge tube with white solid powder), salt bag (for removing moisture and reducing some interferences), and a purification pipe (for further purifying the sample and further reducing the interferences). For solid samples (oyster tissue), each sample (10 g) was placed in an extraction tube and mixed with ultrapure water (5 mL), and then homogenized for 1 min. Subsequently, 15 mL of acetonitrile (containing 1% glacial acetic acid) was added, followed by shaking for 1 min. Then the salt bag was added, followed by shaking for 3 min and centrifuged at 2,930 × g for 5 min (at 4°C). An 5 mL aliquot of the supernatant was transferred into a QuEChERS purification pipe. After shaking for 1 min and concentrating, a 3 mL aliquot was taken and flushed with nitrogen at 45°C to dryness with a nitrogen flushing instrument (Tubes Heater L-129P and Auto-Sample Concentrator L-148, Laiheng, Beijing, China) and redissolved with 2 mL n-hexane. For liquid samples (gastric and intestinal digestive fluids), sample and acetonitrile (with 1% glacial acetic acid) were placed in an extraction tube (v:v = 1:1). For DEL analysis, the gastric and intestinal digestive phases were concentrated 2 and 4 times, respectively, with nitrogen flushing and redissolved with n-hexane as for the solid samples.

#### DEL Determination Using GC-ECD

DEL was determined with an Agilent Technologies 7890B GC coupled to an ECD detector. The quantitative analysis was done on an HP-5 column (30 m × 0.320 mm × 0.25 μm film thickness, Agilent J&W GC columns). The oven temperature program was ramped from 100°C (2 min hold time) to 270°C at 6°C/min with a hold time of 10 min. The injection and detector were at 250 and 300°C, respectively. The nitrogen carrier gas was maintained at a flow rate of 1.0 mL/min. A sample of 1 μL was injected in splitless mode.

Percentages of DEL in the bioaccessible fraction (%) were calculated as follows: BIO × 100/BD, where BIO is the DEL amount detected in the bioaccessible fraction and BD is the DEL amount detected in the same sample before digestion.

### Cell Culture and Transport Assay

Cell culture and transport assay were done using the methodologies previously described by Xu et al. (38) with modification. Briefly, NCM460 cells were spread onto a Transwell polyester permeable membrane support at a density of 1.0 × 105 cells/cm^2^. The cells were grown at 5% CO_2_ and 37°C in a humidified atmosphere in DMEM medium (DMEM: serum:double antibody = 9:1:0.1). The medium was changed every other day to allow the cells to differentiate for at least 21 days. For toxicity assay, cells were incubated with PBS containing DEL (0, 5, 10, 17.5, 30 ng/mL) at 37°C for 24 h. Then, the fluorescence was measured at 450 nm using a Microplate Photometer (Multiskan FC, Thermo Fisher Scientific Inc., Shanghai, China).

The transport assays of DEL were done in a Transwell membrane. NCM460 cell monolayers were incubated with 0.5 mL phosphate-buffered saline buffer (PBS, containing 1 mmol/L Ca^2+^ and 0.5 mmol/L Mg^2+^ ions) containing DEL (0, 2, 10, 30 ng/mL) on the apical side and 1.5 mL PBS on the basolateral side of the monolayers for 2 h and culture medium collected from both sides for GC-ECD. The transepithelial electrical resistance (TEER) of the monolayer was measured to ensure its value was >400 Ω/cm^2^.

### Statistical Analysis

Statistical analysis was done using the IBM Statistical Package for the Social Sciences, SPSS Statistics 26 (IBM Corp., Armonk, NY, USA). One-way ANOVA followed by Duncan's multiple range tests were used to determine the statistical significance. Spearmans' correlation (non-parametric bivariate correction) was established between nutritious substance and DEL levels. Differences were considered significant at *p* < 0.05.

## Results

### Toxin Analysis of DEL in Different Tissues of Oyster

**Figure 2:**
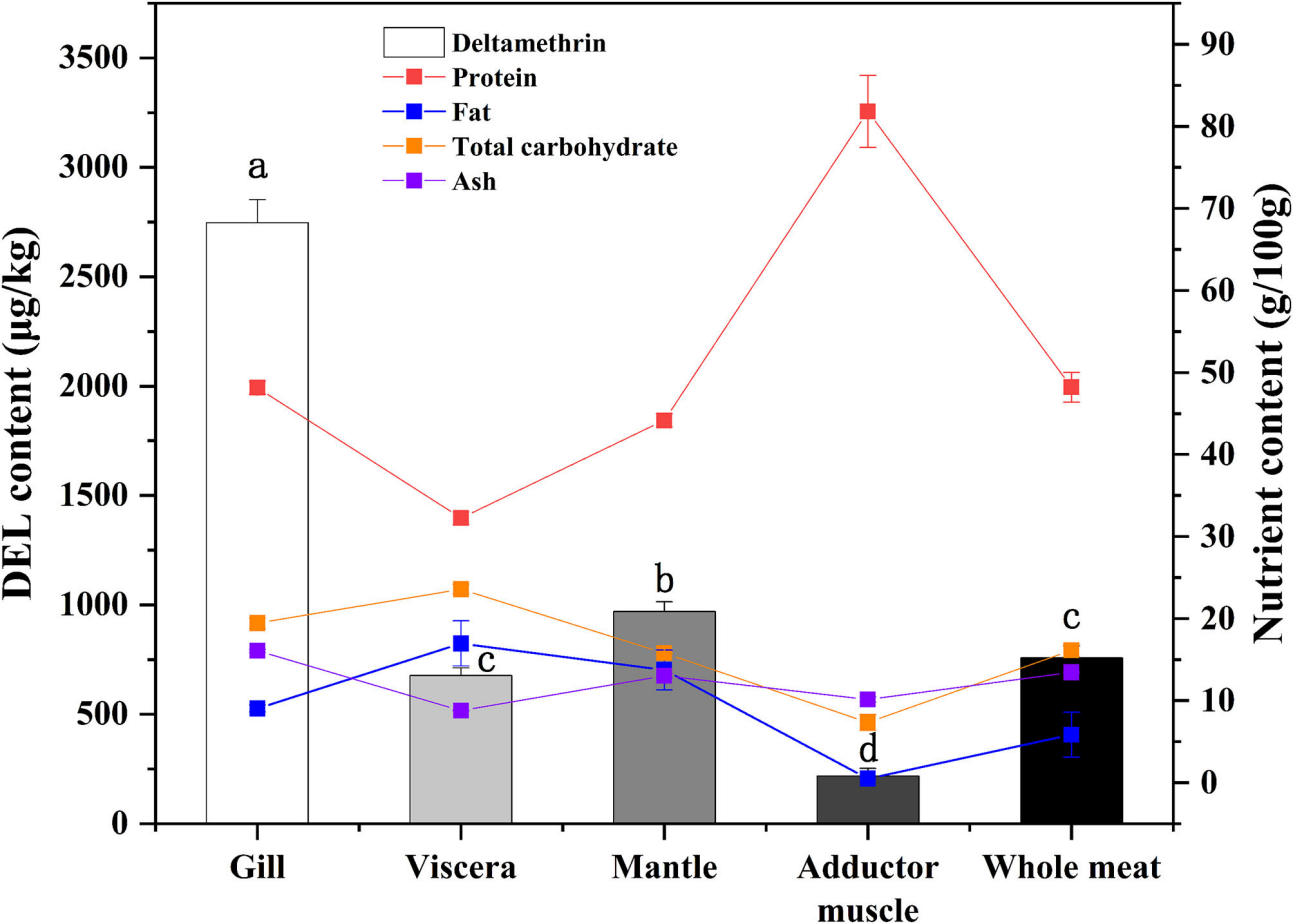
Concentrations of DEL and proximate composition in different tissues of oyster. Lowercase letters represent differences in DEL concentration between tissues (gill, viscera, mantle, muscle, and whole meat) of oyster (ANOVA, *p* < 0.05).

DEL levels in different oyster tissues are shown in Figure 2. DEL toxicity ranged from 677 to 2,750 μg /Kg in different tissues of oysters. The gill sample had the highest levels of DEL. The lowest DEL concentrations were in the adductor muscle samples. The concentration of DEL in the mantle was higher than in the viscera. Statistically significant differences (*p* < 0.05) were observed in the concentration of DEL in the different tissues. The content of DEL in the whole oyster had no significant difference with the content in viscera.

In addition to DEL, the proximate composition (crude protein, fat, total carbohydrate, and ash) in various tissues were also measured (Figure 2). The highest protein content and the lowest fat content were found in adductor muscle. A statistical correlation of DEL levels and tissue proximate composition gave a positive (*r* = 0.729) and highly significant correlation with ash content. No significant correlations were observed between DEL and crude protein (*r* = −0.200, *p* = 0.475), fat (*r* = 0.318, *p* = 0.248) and carbohydrate (*r* = 0.325, *p* = 0.237).

### Bioaccessibility of DEL With Different Cooking Methods

**Figure 3:**
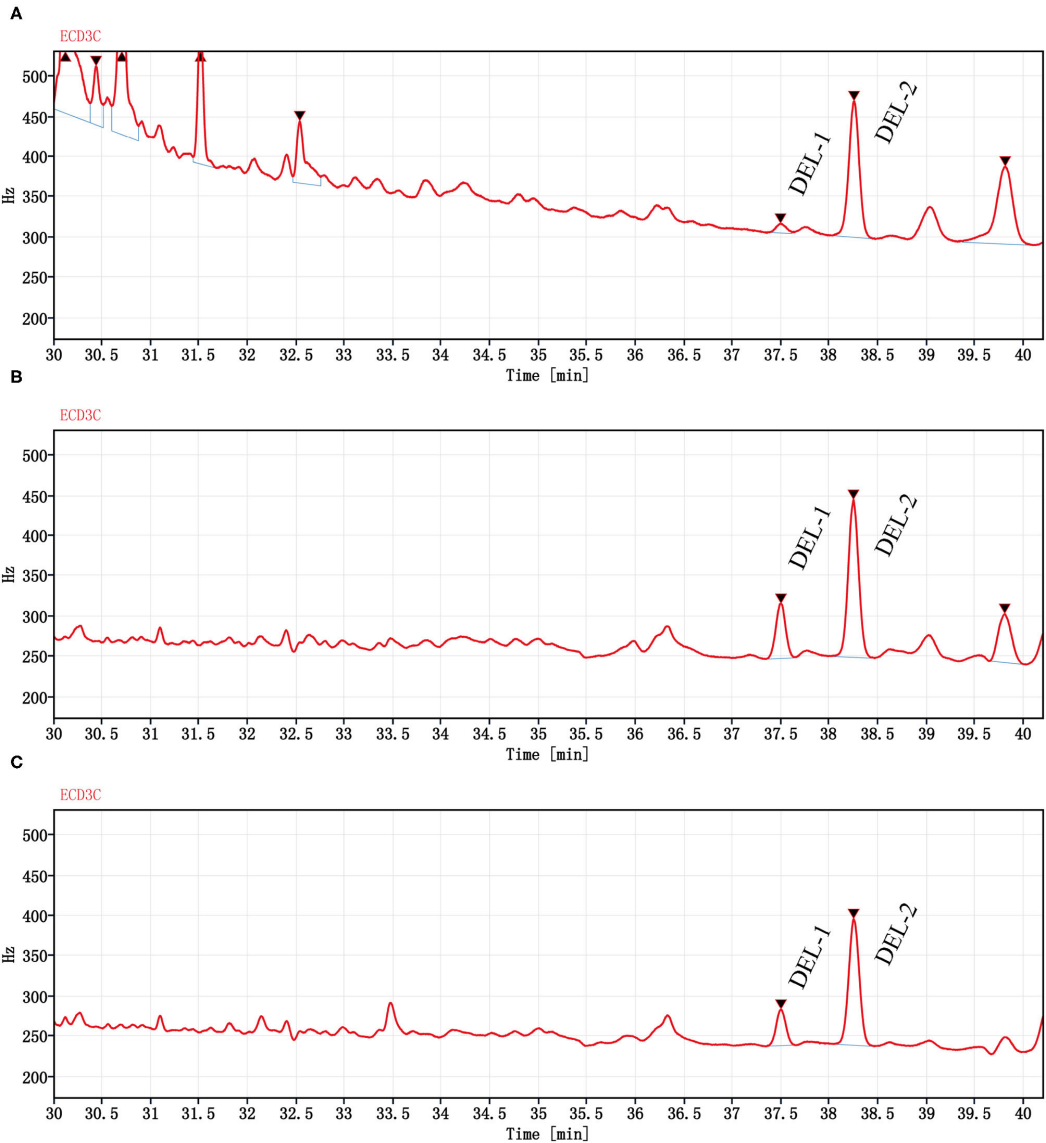
Chromatograms (GC-ECD) of DEL from oyster meat with different processing methods. Chromatograms of DEL are separately in **(A)** Raw oyster; **(B)** Steamed oyster; and **(C)** Roasted oyster. DEL retention time was 37.5 (DEL-1) and 38.2 min (DEL-2). Peak areas of DEL-1 and DEL-2 were 85.4 and 1,280 for raw oysters, 483 and 1,480 for steamed oysters, and 312 and 1,210 for roasted oysters, respectively.

The DEL GC spectrum from oyster meat is shown in Figure 3. There were 2 peaks with retention times of 37.5 min (DEL-1) and 38.2 min (DEL-2). However, there were significant differences between the peak areas of DEL-1 and DEL-2 in oyster meat with different processing methods, and the proportions of DEL-1 was 6.2% (raw), 24.6% (steamed), and 20.4% (roasted).

**Figure 4:**
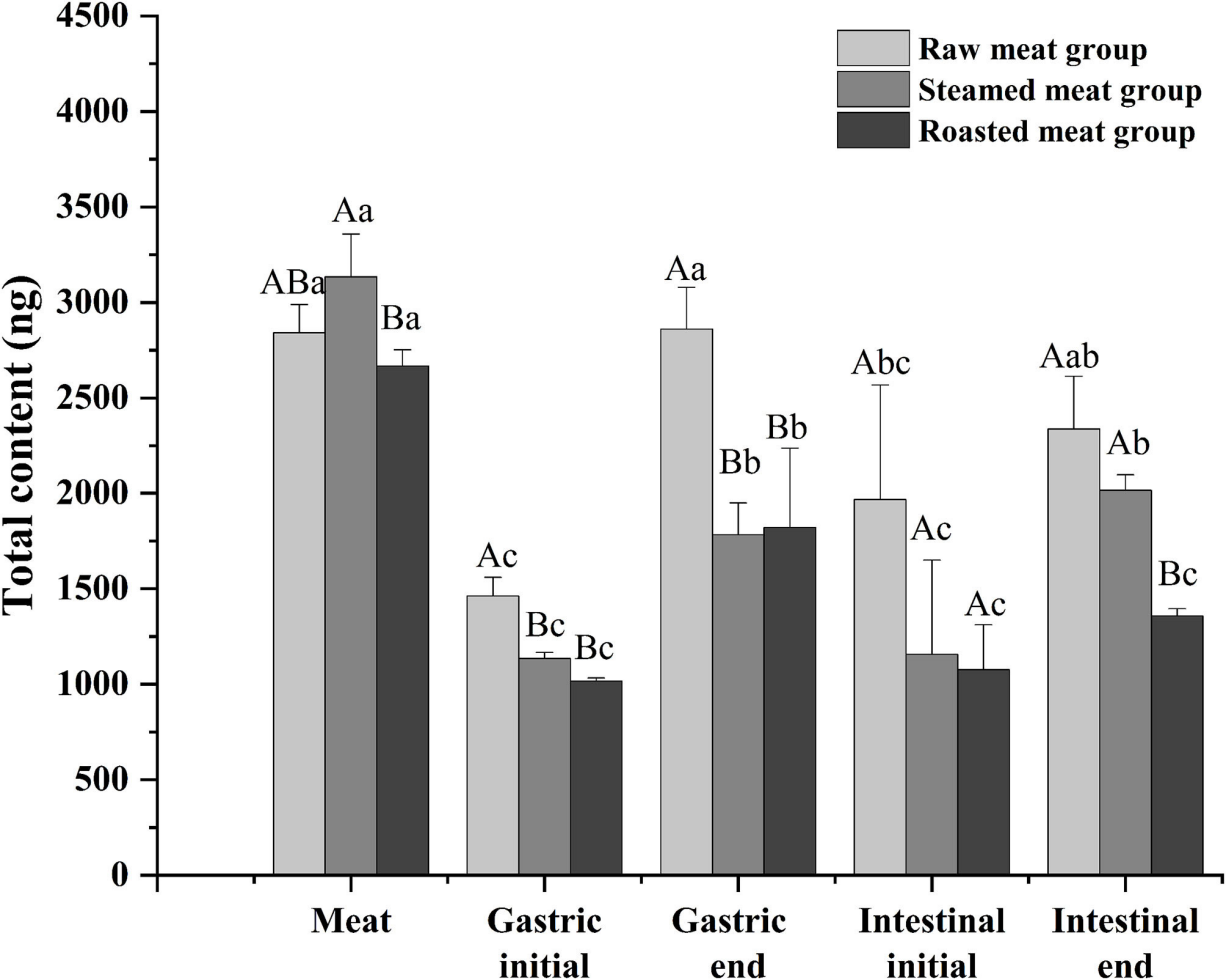
Total content of DEL of oyster meat using three cooking methods at different stages of digestion. Uppercase letters represent differences of DEL content in each digestion stage between different cooking methods (ANOVA, *p* < 0.05). Lowercase letters represent differences of DEL in the digestion process between the same cooking methods (ANOVA, *p* < 0.05).

Total DEL of oyster meats with the various digestion phases (gastric initial and final and intestinal initial and final) with the different cooking methods are shown in Figure 4. There was a similar trend among the three processing methods. The content of DEL at the end of the gastric phase was higher than that at the beginning of the gastric phase, and the content of DEL at the end of the intestinal phase was higher than that at the beginning. The total amount of DEL in the digested liquids was always lower than that in the undigested oyster meat (*p* < 0.05). And the amount of DEL in the raw group was higher than that in the other two groups at each digestive stage. At the end of the simulated digestion, the content of DEL in the raw, steamed, and roasted samples were 2,300 ± 300, 2,020 ± 80, and 1,360 ± 40 ng, respectively.

**Figure 5:**
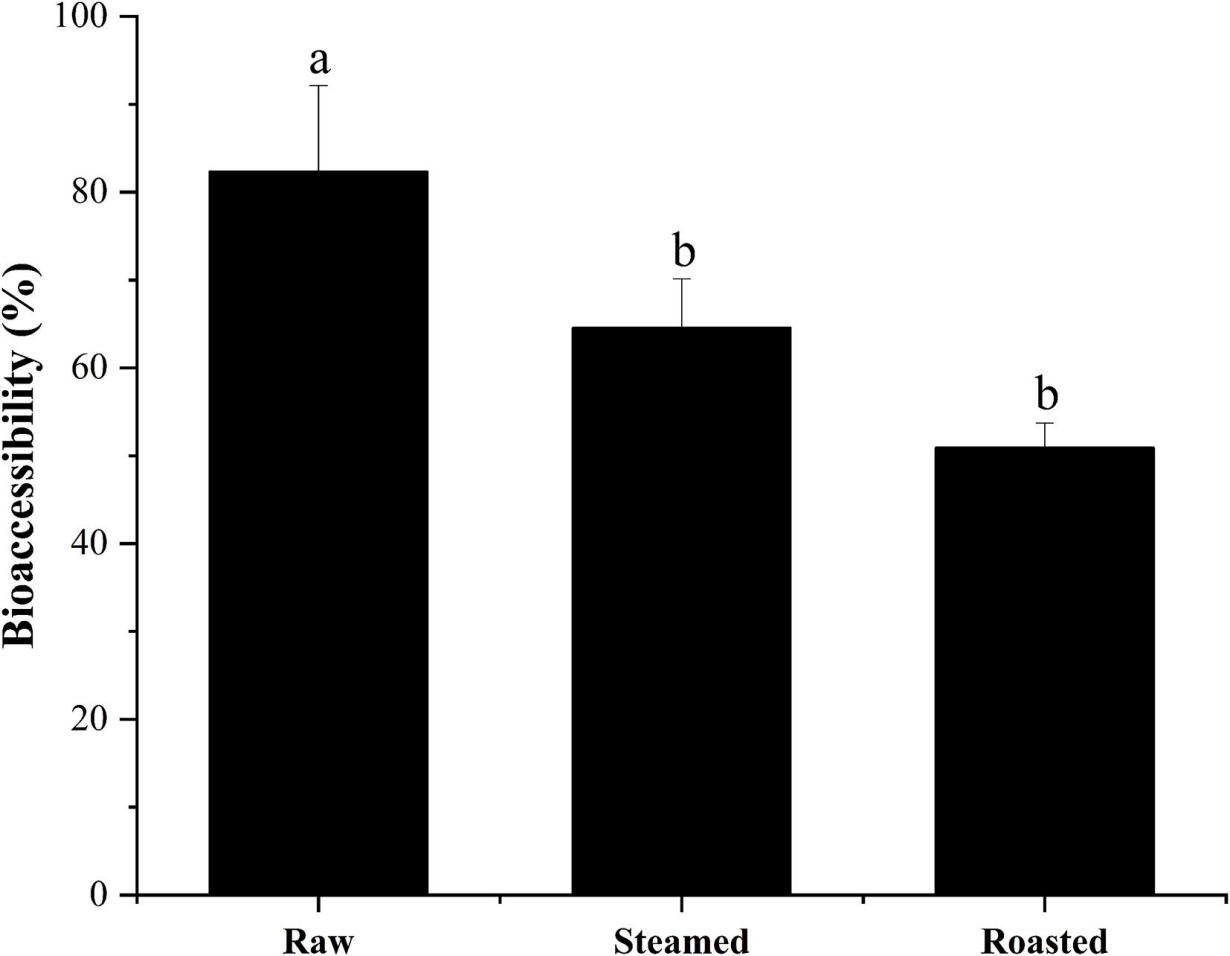
Bioaccessibility (%, mean ± SD) of total DEL content of oyster samples using different cooking methods. Lowercase letters represent differences in bioaccessibility of deltamethrin under different cooking methods (ANOVA, *p* < 0.05).

High percentages of bioaccessible DEL were found with all the cooking methods using the *in vitro* model: 82 ± 10, 65 ± 6, and 51 ± 3% of the total DEL was released into the digestive juice with the raw, steam, and roasting, respectively (Figure 5). There was a significant difference between these cooking methods (*p* < 0.05) and a reduction of bioaccessibility was observed after cooking. No significant difference was found between the steamed and roasted samples, but the bioaccessibility of the roasted group was slightly lower than the steamed group.

### The Change of DEL Concentration During Simulated Digestion

**Figure 6:**
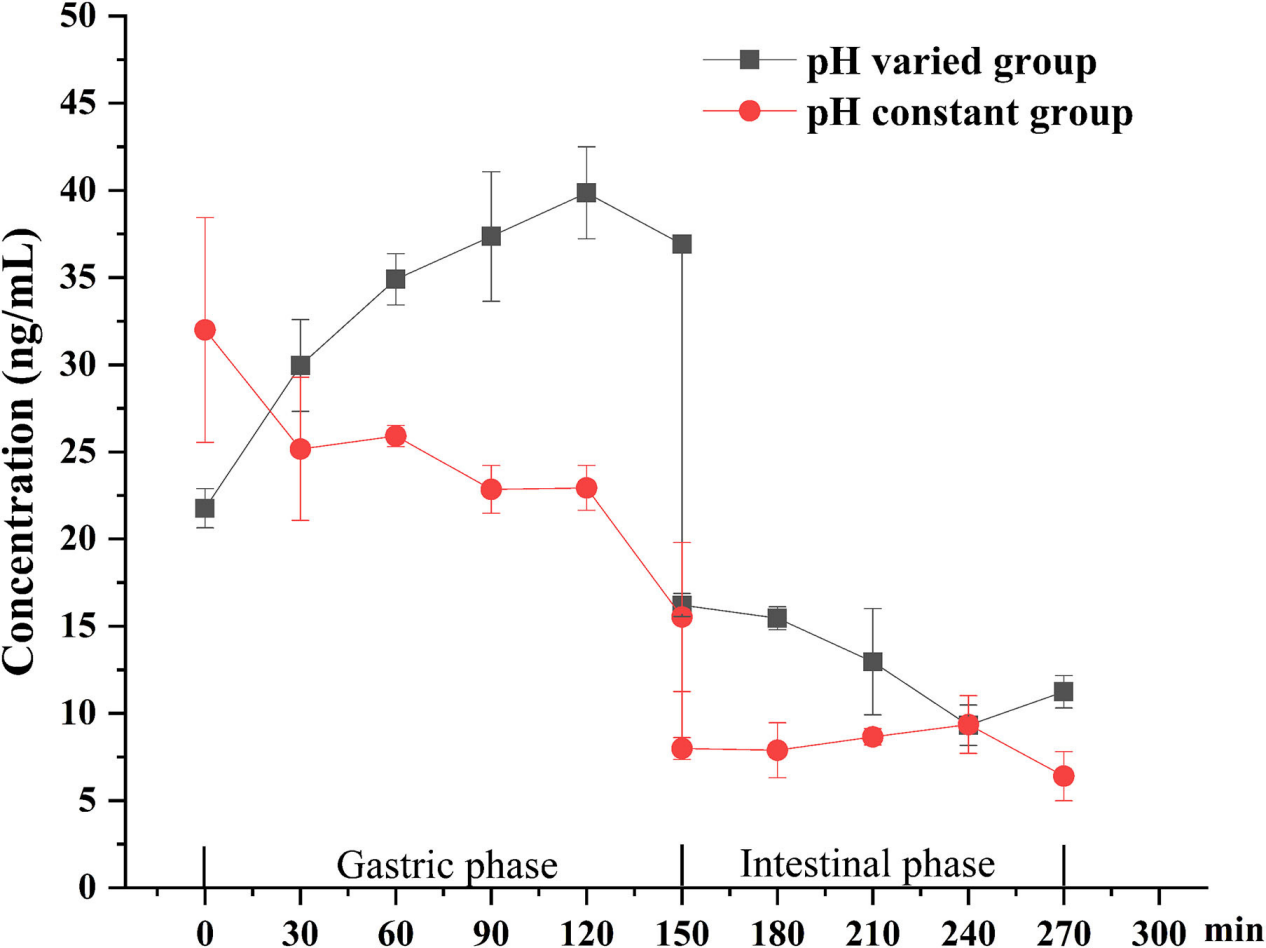
Concentration changes of DEL in digestive juice during simulated digestion with different pH.

The digestion using the different gastric pH vs. a pH constant group. A total of 2 mL HCl was added in the gastric phase. Two hundred microliter HCl was added every 15 min in the pH varied group, and 2 mL HCl was added directly at the beginning of the gastric phase in the pH constant group. As shown in Figure 6, a significant increase in DEL levels was seen in the pH varied group, whereas a significant decrease was observed in the pH constant group during the gastric digestive stage. At the end of the gastric phase, the concentration of DEL in the pH varied group was significantly higher than that in the pH constant group. The switch to intestinal digestion led to an immediate decrease in DEL concentration. At this stage, the concentration of DEL in the pH varied group was higher than in the pH constant group. Furthermore, there were significant differences in the bioaccessibility of DEL between the two groups during gastrointestinal digestion (*p* < 0.05).

### Cellular Absorption of DEL

**Figure 7:**
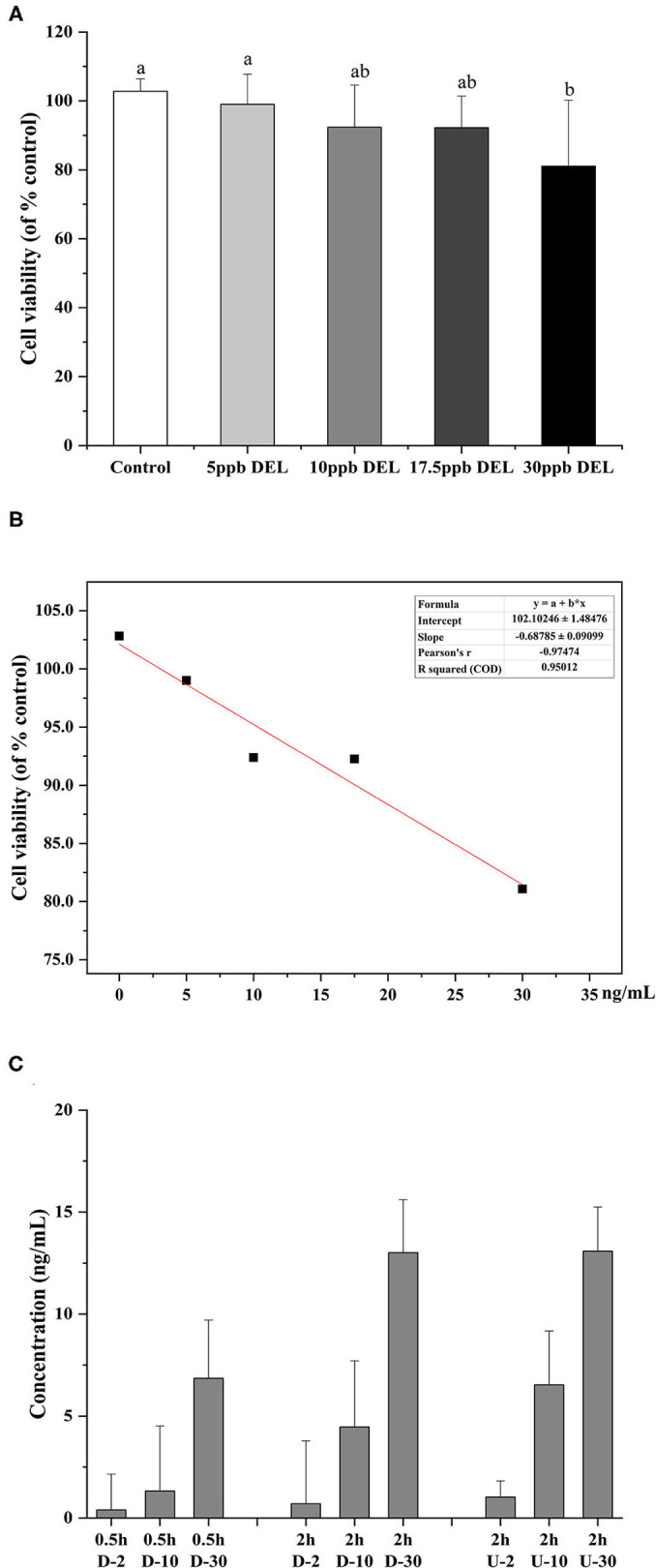
Toxicity of DEL on the viability of NCM460 cells and transport efficiency. **(A)** Effect of DEL on the viability of NCM460 cells. The cell viability was significantly decreased after treatment with 30 mM DEL for 24 h. The data are expressed as the means ± SD (*n* = 9). Values with different letters are significantly different (*p* < 0.05). **(B)** Linear curve of cell viability (% of control) with DEL concentration. **(C)** Transport of DEL across NCM460 cell monolayers. The letter D represents the lower chamber of the Transwell, and the letter U represents the upper chamber of the Transwell. The concentration in the lower chamber shown in the figure is 3 times the actual concentration.

As shown in Figure 7A, after treatment with 5, 10, 17.5, and 30 ng/mL DEL for 24 h, there was no significant difference in the cell viability between control, 5, 10, and 17.5 ng/mL, whereas a significant reduction of cell viability was found at 30 ng/mL (*p* < 0.05). It was assumed that no harm came to the cells used in the transport study because the transport time used was 2 h, much shorter than 24 h for DEL toxicity. Figure 7B shows the linear curve of cell viability with DEL concentration at 24 h. As the concentration of DEL increases, cell viability continues to decrease. The linear equation obtained was y = 102.1–0.69 x, and the Pearson correlation coefficient was −0.97. Transport of DEL across NCM460 cell monolayers is shown in Figure 7C. The concentration of DEL in the 2 h group was slightly higher than that in the 0.5 h group.

The degradation of DEL was not observed within the cell, as evidenced by no significant differences (*p* > 0.05) between the total amount of DEL toxin added and the sum of this toxin found in the apical and the basolateral sections after 2 h.

## Discussion

### Occurrence and Profiles of DEL in Oysters

After a short exposure experiment, the content of DEL was the highest in the gills of oysters, followed by the mantle and viscera, and the lowest in the muscle. The digestive pathway of DEL in oysters is from gills to internal organs and then to muscles. The mantle is a membrane that wraps the internal organs and tissues. The high content of DEL in mantle may be due to the direct contact between the mantle and water, and the lipophilicity of DEL allowed it to be absorbed in the mantle. In addition, the concentration of DEL and the proximate composition in whole meat was basically the same as the average value in each organ. Whole oyster meat is normally consumed. According to the data in Figure 4, there was no significant difference in the total content of DEL in raw, steamed, and roasted oysters. However, the concentration of DEL in steamed and roasted oysters was higher than that in raw oysters because the steaming and roasting process will cause water loss. This was consistent with the studies by Hess et al. (39), who suggested that the concentration of azaspiracids (AZA) in cooked shellfish was 2-fold higher than the uncooked shellfish. And Wiech et al. (40) also show the similar result.

The occurrence and profiles of DEL in oysters may not only be related to the tissue and cooking method, but also related to the structural characteristics of DEL itself. DEL contains 3 chiral centers, indicating that it has 8 stereoisomers (41, 42). Corcellas et al. (43) showed that the cis isomers of pyrethroids were easier to enrich than trans isomers in organisms. But the degradation rate of trans isomers was faster than cis isomers (44). Usually, there were two peaks in the deltamethrin gas chromatogram (45). As shown in Figure 3, there was a higher accumulation of DEL-1 in steamed or roasted oyster compared to raw oyster, whereas DEL-2 was the predominant toxin accumulated. However, the structure of DEL-1 is not understood. DEL-1 and DEL-2 may be two different stereoisomers of DEL and the conversion of DEL-2 to DEL-1 may have occurred due to heating. Therefore, more research regarding enantioselective accumulation and enantiomeric toxicology is needed to establish which enantiomers have a greater health risk.

### DEL Bioaccessibility and Changes After *in vitro* Digestion and Cell Monolayers

Compared to other pyrethroids, DEL has a higher toxicity, which makes it important to study its bioaccessibility. The *in vitro* digestion model indicated that the DEL was gradually released from oysters during digestion. The simulated digestion, showed a lower concentration of DEL with intestinal digestion than in the prior gastric digestion. This may be due to the dilution of DEL by intestinal digestive juice such as bile.

The results observed in the present study show that DEL bioaccessibility in steamed oysters was around 65%, and in roasted oysters was around 51%, which is significantly lower than in raw oysters (82%). These results were consistent with the previous observations that the bioaccessibility values of AZA were lower in mussels after steaming (46). The hydrophobic peptides become exposed with surface denaturation. Since AZA are lipophilic, a weak binding between AZA and a 45 KDa protein was observed during cooking. DEL is also lipophilic, and this binding force might explain the lower bioaccessibility after heating. Considering the high bioaccessibility, some studies have shown that the content of harmful substances in food can be reduced by washing and soaking (47). Before eating oysters, we can try to reduce the harm of harmful substances to human body by washing and soaking.

The DEL content decreased significantly after intestinal digestion, which might be explained two ways. One is that DEL was present in the biologically unavailable parts (materials not digested), and the other is that the presence of pancreatin and bile salts in the digestive juice of the intestine hydrolyzed or degraded DEL. Previous studies showed that some pyrethroids including DEL may undergo significant transformations in the intestinal fluid (48). Carboxyl ester lipase has an important role in lipid metabolism and is synthesized primarily in the pancreas (49). Crow et al. (50) also observed a correlation between carboxylesterase and pyrethroids, indicating trans-permethrin were effectively cleaved, while DEL and bioresmethrin were not metabolized. The degree of hydrolysis varied for different pyrethroids. Carboxylesterase may therefore hydrolyze pyrethroids during simulated digestion *in vitro*. The degree of hydrolysis may also be related to the concentration of carboxylesterase. And the toxicity of the hydrolysate may be higher or lower than that of its parent compound (17). Thus, the hydrolytic metabolites and their potential biological activity should be investigated in future research.

The difference between the *in vitro* simulated digestion model and the existing static *in vitro* model is mainly reflected in the pH of gastric phase. The importance of pH values with *in vitro* simulated digestion in the bioaccessibility has been previously suggested (51, 52). Gastric pH is not a constant parameter and its value changes continuously during digestion (37). The concentration of DEL in the constant pH group was lower than the variable pH group for most stages of digestion as was its bioaccessibility.

*In vitro* simulated digestion experiments are simpler and more convenient than *in vivo* experiments partly because only specific enzymes are used *in vitro*. There are many *in vitro* digestion models, and the bioaccessibility data obtained by different digestion models may be different (53). Other factors when studying the bioavailability of pyrethroids such as the diversity of small intestinal microbiome and microbial diversity, as well as other enzymes and biosurfactants in digestive juices should be considered (48).

To reduce the influence of the complex components in the simulated digestive juice on the cell viability during cell transport, a standard solution of DEL with a similar concentration instead of intestinal digestion chyme was used. The transmembrane transport efficiency of DEL by NCM460 cells were 35, 45, and 43% at 2, 10, and 30 ng/mL, respectively. After simulated digestion, the concentration of DEL was about 10 ng/mL. Using a trans-transport efficiency of 45%, the bioaccessibility of DEL after passing through small intestinal epithelial cells were 37% (raw), 29% (steamed), and 23% (roasted). Shellfish like oysters, most often consumed raw, are the most hazardous, while foods consumed soon after heat treatment have fewer hazards (25).

The bioaccessibility of DEL with simulated digestion should help provide further information for the risk assessment of pyrethroids. However, there are some differences between bioavailability and bioaccessibility. The NCM460 cells were was used to study bioaccessibility, but the relationship between it and bioavailability still needs further study.

## Conclusions

The results should help to evaluate potential dietary exposure to some pesticide toxins through the consumption of seafood. The bioaccessibility of DEL from raw, steamed, and roasted oyster samples were 82, 65, and 51%, respectively, during gastrointestinal digestion. Roasting is the recommended method of oyster cooking to lower DEL bioaccessibility.

## Data Availability Statement

The raw data supporting the conclusions of this article will be made available by the authors, without undue reservation.

## Author Contributions

YJ preformed conceptualization, methodology, software, investigation, and wrote the original draft. CL was responsible for validation, formal analysis, visualization, and software. CF participated in the design of simulated digestion method. YT, YL, and JR was responsible for writing - review and visualization. HH contributed project administration, funding acquisition, supervision, and resources. All authors discussed the results, contributed to the final manuscript, approved the final version of the manuscript, and agree to be accountable for the study.

## Conflict of Interest

The authors declare that the research was conducted in the absence of any commercial or financial relationships that could be construed as a potential conflict of interest.

## Publisher's Note

All claims expressed in this article are solely those of the authors and do not necessarily represent those of their affiliated organizations, or those of the publisher, the editors and the reviewers. Any product that may be evaluated in this article, or claim that may be made by its manufacturer, is not guaranteed or endorsed by the publisher.
